# Genetic assignment of illegally trafficked neotropical primates and implications for reintroduction programs

**DOI:** 10.1038/s41598-020-60569-3

**Published:** 2020-02-28

**Authors:** Luciana Inés Oklander, Mariela Caputo, Agustín Solari, Daniel Corach

**Affiliations:** 1 Grupo de Investigación en Genética Aplicada (GIGA), Instituto de Biología Subtropical (IBS), Nodo Posadas, Jujuy 1745, N3300NFK Posadas, Universidad Nacional de Misiones (UNaM) – CONICET, Misiones, Argentina; 2 Universidad de Buenos Aires, Facultad de Farmacia y Bioquímica, Departamento de Microbiología Inmunología Biotecnología y Genética, Cátedra de Genética Forense y Servicio de Huellas Digitales Genéticas, Junín 956, C1113AAD Buenos Aires, Argentina; 3 CONICET - Consejo Nacional de Investigaciones Científicas y Tecnológicas, C1033AAJ Buenos Aires, Argentina; 4 Instituto de Biología Subtropical (IBS), Universidad Nacional de Misiones (UNaM) – CONICET, Nodo Iguazú, Bertoni 68, 3370, Puerto Iguazú, Misiones, Argentina

**Keywords:** Population genetics, Ecological genetics

## Abstract

The black and gold howler monkey (*Alouatta caraya*) is a neotropical primate threatened by habitat loss and capture for illegal trade in Argentina. Using multilocus microsatellite genotypes from 178 *A. caraya* individuals sampled from 15 localities in Argentina, we built a genotype reference database (GRDB). Bayesian assignment methods applied to the GRDB allowed us to correctly re-assign 73% of individuals to their true location of origin and 93.3% to their cluster of origin. We used the GRDB to assign 22 confiscated individuals (17 of which were reintroduced), and 3 corpses to both localities and clusters of origin. We assigned with a probability >70% the locality of origin of 14 individuals and the cluster of origin of 21. We found that most of the confiscated individuals were assigned to one cluster (F-Ch-C) and two localities included in the GRDB, suggesting that trafficked *A. caraya* primarily originated in this area. Our results reveal that only 4 of 17 reintroduced individuals were released in sites corresponding to their cluster of origin. Our findings illustrate the applicability of genotype databases for inferring hotspots of illegal capture and for guiding future reintroduction efforts, both of which are essential elements of species protection and recovery programs.

## Introduction

Similar to other countries, wildlife illegal hunting and trade are threats to Argentinian wildlife. Confiscated and surrendered animals from trafficking are transported to rehabilitation centres, and the return of these confiscated animals to the wild receives strong support from the public. Ideally, trafficked animals would be reintroduced into the population that they were extracted from or translocated to another suitable site within the species’ original range. Although translocations are considered a good option for conservation and a solution to trafficked animals^1^, they might be detrimental for the animal and/or the environment if choices over where to return individuals to their natural habitats are not properly based on scientific evidence^2–4^. For example, when significant genetic structure exists within the species in question, translocations may inadvertently lead to admixture of distinct evolutionary lineages and act to homogenize existing diversity and biogeographic patterns instead of protect them^5^. However, the genetic consequences of translocations have seldom been studied^6^, and in Latin America, the development of biodiversity management and conservation plans as part of public policy have not yet taken advantage of newly developed genetic techniques to inform translocation policy and decisions^7^. Conservation genetics can help strengthen the links between scientists and decision makers and improve reintroduction and translocation policy.

Molecular genetic studies using individuals of known origin allow researchers to calculate levels of differentiation among populations and to assess population structure within a species in the wild^8–10^. Based on this, the populations studied may be grouped into clusters based on genetic similarity. Once such an assessment of differentiation between populations/localities or clusters is obtained, genetic assignment analysis of individuals of unknown provenience can be performed to identify the likely locality or cluster to which they belong – i.e., to infer their geographical sites of origin. This identification, made in advance of any translocation attempt, enables researchers to estimate the genetic diversity introduced into the environment when translocations and/or reintroductions occur. Genetic assignment to a geographic area of origin is possible only when a suitable genotype reference database (GRDB) is available to compare results. The construction of a GRDB represents a requirement and, ideally, should include the largest possible sample across a species’ distribution range. Moreover, the data quality must be extremely reliable, because future determinations will rely on these data.

Geographic origin determination using simple tandem repeats markers (STRs, also known as “microsatellites”) has already been applied to several species, such as bobcats^11^, tortoises^12^, African elephants for the ivory trade^13,14^, mouflons^15^, bears^16^, salmon^17^, timber^18^, palm trees^19^, and macaws^10^. These studies allowed researchers to: (1) accurately discriminate among ivory trafficked from elephants from the four major regions of Africa^13,14^, (2) identify poaching of the protected Sardinian mouflon, where a suspected poacher found in possession of a carcass initially claimed that it was from a sheep from his flock^15^, (3) identify farmed Atlantic salmon escapes representing a threat to the genetic integrity of natural populations^17^, (4) develop a genetic reference database characterising the populations of origin for illegally logged timber, which creates market disadvantages for products from sustainable forestry^18^, (5) determine that illegally-traded palm species in Brazil were obtained from several sites showing that there is no single target locality used by poachers^19^, and (6) differentiate among macaws from 3 geographic regions of Brazil and identify the most probable regions of origin of 24 confiscated individuals^10^. These approaches underscore the importance and potential of molecular analyses for wildlife management and for identification of species to prevent illegal trafficking.

*Alouatta caraya* is the most commonly trafficked primate species found in the illegal pet trade in Argentina. Trade in this species appears to be internal as there are no records of cross-national trafficking. This trafficking is reflected by the number of individuals confiscated during control and inspection operations^20–22^. Official records from the National Wildlife Surveillance and Control Directorate show that 10 individuals of *A. caraya* were confiscated and/or voluntarily surrendered in the last 4 years, although the number of unofficial cases is higher. *Alouatta caraya* is globally categorized as “Near Threatened” by the IUCN^23^, but is considered “Vulnerable” in the red list of Argentina^24^ due to population reduction, decreased area of occupancy and/or quality of habitat, increased exploitation due to hunting or illegal traffic (pet trade), and the effects of pathogens and parasites. Currently, Argentina’s native forests are rapidly being replaced as a result of growing urbanization, industrialization, and large-scale clearing for agricultural purposes^21,22^. This process of transforming native subtropical forests is clearly evident in northern Argentina where five species of primates are found (*Alouatta caraya, Alouatta guariba clamitans, Aotus azarai, Sapajus nigritus* and *Sapajus cay*). Of these five, both *Alouatta* species are of great epidemiological importance since they are highly sensitive to the Yellow Fever virus (YFV). *Alouatta caraya* show high mortality when infected by the virus. Thus, this species serves as an early epidemiological sentinel, allowing the establishment of control and prevention measures^24–27^.

**Figure 1:**
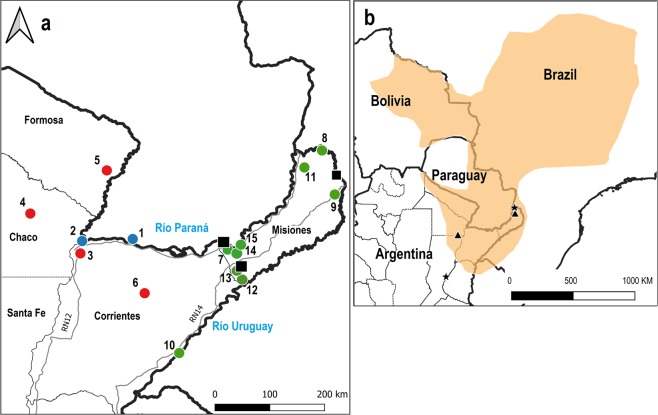
(**a**) Map of the 15 localities included in the database of *A. caraya* genotypes in Argentina. Maps show (1 to 10) previously published and (11 to 15) newly sampled localities. Color-coded circles indicate the three genetic clusters identified using the structure analysis in the present study: blue: P-RP cluster, red: F-Ch-C cluster, and green: M-RU cluster. The complete names of sampling sites are listed in Table 1. Black squares indicate the sites were corpses of *A. caraya* were found. (**b**) Map showing the distribution range of *A. caraya*. Black stars show the location of the rescue centres included in this study. Black triangles represent the reintroduction sites.

*Alouatta caraya* has been the focus of several population genetic studies of dispersal patterns, kinship, reproductive success, and phylogeography, among other topics^28–31^. In a recent study, ten nuclear microsatellites were used to generate a DNA genetic reference database characterising the southernmost populations of this species^9^ (Fig. 1). Genetic clustering of the studied populations identified a number of distinct genetic groups considered as clusters or management units (MUs)^9^. One of these clusters consists of populations inhabiting different localities in the Atlantic Forest in the littoral zone bordering Brazil (Misiones cluster^9^). As part of the monitoring program for the epidemiological surveillance of YFV and other arboviruses in non-human primates (NHP), the number of genotypes included in the reference database for this area was increased with samples collected during 2017 and 2018.

Here, we present the results of genetic analyses designed to estimate the origin of 22 *A. caraya* confiscated from the illegal trade and subsequently housed at the Güirá-Oga rescue centre (Güirá-Oga) in Puerto Iguazú, Misiones and the rescue centre Estación Zoológica Experimental Granja La Esmeralda (Esmeralda) in Santa Fe, Argentina, as well as 3 individuals found dead in cities in northern Argentina. We aimed to infer the geographic site of origin of these animals by comparing the specimens’ genetic profiles with the profiles included in a regional GRDB for *A. caraya*.

This study underscores the use of STR-based genetic databases as a tool for identifying the origin of individual animals, an outcome with application to understanding patterns of illegal wildlife trade, for assessing whether prior translocations in northern Argentina had indeed occurred into likely areas of origin, and to provide a reliable tool for future translocation/reintroduction program protocols.

## Results

Genotypes of 143 individuals from 10 localities (Locs) were available from previous studies^9,28^. To these, we added newly derived and complete genotypes at 10 microsatellite loci obtained from fecal samples of 39 individuals from 5 new localities. Two fecal samples were collected from each of these individuals, and the genotype assignment for each individual at each locus was replicated twice (in the case of heterozygotes) or four times (in case of homozygotes) in order to minimize possible genotyping errors due to allelic dropout. We likewise constructed complete 10-locus genotypes for all 25 additional individual samples of unknown provenience for genetic assignment. The complete Genotype database obtained from all localities and clusters of *A. caraya* studied in this investigation is available at: 10.5281/zenodo.3660723, and the genotypes of all the assigned individuals are presented as Supplementary Table S1.

We did not observe evidence of scoring errors due to stuttering, large allele dropout, or null alleles for any locus in any population in our screening with the program Micro-Checker v2.2.3^32^.

Using Arlequin v 3.5^33^, we did not observe evidence of linkage between any pair of loci (P > 0.05). Significant deviation from Hardy-Weinberg equilibrium was only detected by GenAlEx v 6.5^34^ and Arlequin v 3.5^33^ softwares for the marker D8S165 in Loc 9 (Piñalito Province Park). Significant evidence of inbreeding (inbreeding coefficient: F_IS_ = 0.29) was already found for this population in a previous study^9^. The numbers of different alleles, effective and private alleles, observed heterozygosity (H_o_), expected heterozygosity (H_e_) and unbiased expected heterozygosity (uH_e_) are presented in Table 1.

**Table 1:** Total sampling for IGDB for *A. caraya* in Argentina.

Locality number	Locality name	Geographic coordinates		N samples	Na		Ne		He		uHe		AR		PA	FIS
					Average	SD	Average	SD	Average	SD	Average	SD	Average	SD		
1	Paraguay	−27.275	−57.684	5	2.800	0.442	2.038	0.304	0.420	0.072	0.467	0.080	3.082	0.925	1	0.179
2	Isla, Rio Paraná	−27.314	−58.646	36	4.800	0.862	2.550	0.411	0.501	0.080	0.508	0.081	2.449	0.969	2	0.012
3	EBCO, Corrientes	−27.550	−58.679	40	4.500	0.934	2.165	0.339	0.440	0.078	0.445	0.079	2.619	1.096	2	−0.039
4	PN Chaco, Chaco	−26.794	−59.618	9	2.900	0.277	1.813	0.154	0.410	0.051	0.435	0.054	2.779	1.006	1	0.084
5	Guaycolec, Formosa	−25.985	−58.161	12	3.700	0.539	2.285	0.271	0.495	0.067	0.517	0.070	2.211	1.095	1	0.080
6	San Alonso, Corrientes	−28.306	−57.456	10	2.700	0.473	1.914	0.310	0.356	0.090	0.374	0.095	2.500	0.707		−0.140
7	Garupa, Misiones	−27.467	−55.827	6	2.800	0.359	1.896	0.290	0.385	0.072	0.420	0.079	2.738	0.959		−0.169
8	Yacutinga Lodge, Misiones	−25.574	−54.075	6 (2 Ni)	2.400	0.427	1.877	0.312	0.376	0.070	0.411	0.077	2.200	0.789	1	−0.198
9	PP Piñalito, Misiones	−26.500	−53.833	11 (3 Ni)	4.400	0.521	2.591	0.301	0.551	0.065	0.577	0.068	2.848	0.822	4	0.204
10	Yapeyu, Corrientes	−29.445	−56.800	9	3.600	0.306	2.414	0.300	0.509	0.072	0.539	0.076	2.784	0.817		−0.056
11	PP Lago Urugua-í, Misiones	−25.921	−54.419	9	3.500	0.522	2.359	0.333	0.504	0.068	0.533	0.072	2.178	1.032		0.080
12	Azara, Misiones	−27.984	−55.787	5	2.500	0.269	1.841	0.194	0.394	0.068	0.438	0.076	2.773	1.121	1	−0.271
13	Apóstoles, Misiones	−27.910	−55.761	4	2.500	0.224	1.865	0.121	0.441	0.041	0.504	0.047	2.593	1.186		−0.169
14	Reserva Urutau EBY	−27.518	−55.788	4	2.200	0.249	1.731	0.180	0.359	0.071	0.411	0.081	2.336	0.748		−0.113
15	Sta Cecilia, Misiones	−27.429	−55.710	12	3.800	0.512	2.330	0.218	0.522	0.064	0.545	0.066	2.347	0.555	2	0.085

Localities sampled in Oklander *et al*.^9^ plus samples collected during the monitoring program for the epidemiological surveillance of YFV and other arboviroses in NHP in 2017 and 2018. N samples: number of individuals sampled in each population; Na: N° of different alleles; Ne: N° of effective alleles (calculated as 1/∑(allele frequency)^2^); He: expected heterozygosity = 1 − ∑(allele frequency)^2^; uHe: unbiased expected heterozygosity = (2N/(2N − 1))*He; AR: allelic richness; PA: N° of alleles unique to a single population; FIS: inbreeding coefficient: Ni: New individuals obtained in resampled localities.

**Figure 2:**
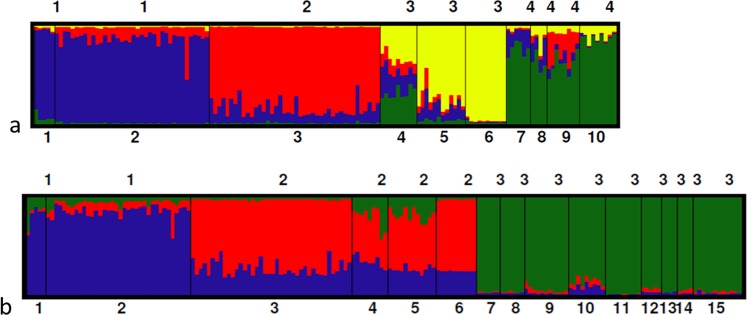
(**a**) Structure analysis of clusters in *A. caraya*^9^ (*K* = 4): blue: P-RP cluster 1, red: EBCO cluster 2, yellow: F-Ch-C cluster 3, and green: M-RU cluster 4. (**b**) Structure analysis incorporating samples from the new localities (11 to 15) (*K* = 3) sampled in the present study: blue: P-RP cluster 1, red: F-Ch-C cluster 2, and green: M-RU cluster 3. Individuals are represented by vertical lines (y-axis) coloured in proportion to their membership coefficients in each cluster and grouped into populations of samples and separated with a black line. Complete names of populations are listed in Table 1.

Analysis using the software Structure v.2.3.4^35^ and applying the method described by Evanno^36^ to a dataset comprising genotypes already collected for *A. caraya* in Oklander *et al*.^9^ plus the new localities sampled here showed that the most likely number of different genetic clusters was three (K = 3, Fig. 2). This new analysis resulted in the disappearance of one previously published cluster (EBCO cluster^7^, K = 4, Fig. 2), which in our expanded dataset now clusters with the localities of Chaco National Park, Chaco (Loc 4), Guaycolec, Formosa (Loc 5), and San Alonso, Corrientes (Loc 6, Fig. 2). Accordingly, the complete set of 15 locations sampled were grouped into 3 distinct clusters or regions (Fig. 1). Cluster 1 includes two localities (Locs 1 and 2) from Paraguay-Isla Rio Paraná (P-RP); cluster 2 includes four localities (Locs 3 to 6) from Formosa-Chaco-Corrientes (F-Ch-C); and finally, cluster 3 includes nine localities (Locs 7 to 15) from Misiones-Rio Uruguay (M-RU).

Aiming to evaluate the efficiency of the software GeneClass2^37^ for assigning individuals to their localities or clusters of origin, we first tested the individuals included in the complete database whose origins was known. The software correctly assigned 73% of individuals in the database (Quality index 68.73%) when separated according to the 15 localities and 93.3% (Quality index 89.23%) when separated according to the three clusters. We then used GeneClass2 to assign the genotypes of each of the 22 confiscated individuals and 3 corpses of unknown origin to both localities and clusters in the GRDB (Table 2). We established a threshold value to consider the assignment to be reliable if it was higher than 70%^38,39^ (Table 2).

**Table 2:** Genetic assignment of individuals using GeneClass2 and according to the criteria described by Rannala & Mountain^57^ within the 15 localities that compose the database of genotypes for *A. caraya* in Argentina (column 1–2), and within the 3 genetic clusters identified for *A. caraya* in Argentina (column 3–4).

	Best matching population	Score %	Best matching cluster	Score %
Güirá-Oga 1	Loc 6	96.46	F-Ch-C	86.07
Güirá-Oga 2	Loc 5	84.56	F-Ch-C	99.84
Güirá-Oga 3	Loc 3	44.28*	F-Ch-C	98.38
Güirá-Oga 4	Loc 2	75.75	P-RP	93.51
Güirá-Oga 5	Loc 7	38.42*	M-RU	96.57
Güirá-Oga 6	Loc 3	39.59*	F-Ch-C	75.37
Güirá-Oga 7	Loc 3	49.6*	F-Ch-C	70.25
Güirá-Oga 8	Loc 3	98.06	F-Ch-C	99.98
Güirá-Oga 9	Loc 5	70.05	F-Ch-C	100
Güirá-Oga 10	Loc 2	98.99	P-RP	99.59
Güirá-Oga 11	Loc 2	44.47*	P-RP	99.31
Güirá-Oga 12	Loc 3	49.41*	F-Ch-C	86.05
Güirá-Oga 13	Loc 3	96.65	F-Ch-C	93.83
Güirá-Oga 14	Loc 3	99.81	F-Ch-C	96.88
Güirá-Oga 15	Loc 3	96.54	F-Ch-C	88.15
Güirá-Oga 16	Loc 13	74.15	M-RU	99.97
Güirá-Oga 17	Loc 1	89.15	P-RP	99.87
Esmeralda 1	Loc 10	53.12*	M-RU	99.37
Esmeralda 2	Loc 3	89.16	F-Ch-C	81
Esmeralda 3	Loc 3	62.74	F-Ch-C	56.68**
Esmeralda 4	Loc 3	98.56	F-Ch-C	97.92
Esmeralda 5	Loc 6	74.28	F-Ch-C	99.15
Found dead in Loc 13, Misiones	Loc 5	80.29	F-Ch-C	98.04
Found dead in Posadas, Misiones	Loc 15	54.69*	M-RU	69.54**
Found dead in San Antonio, Misiones	Loc 12	42.38*	M-RU	99.67

Threshold: 0.05. *Individuals values below the threshold for location assignment. **Individuals values were below the threshold for cluster assignment.

For the 25 individuals analysed, 9 individuals’ assignment values were below the threshold for assignment to a specific locality. The lack of assignment was expected because not all known populations of *A. caraya* are represented in the database.

Of the 15 confiscated individuals with assignment values above the threshold, almost half of them (seven) most likely came from Loc 3, two from Loc 2, two from Loc 5, two from Loc 6, one from Loc 1 and one from Loc 13. Additionally, of the 3 corpses found in cities, we could only assign one them, found in Loc 13, to Loc 5, therefore showing a different likely site of origin from the place where was found (Table 2).

As a means to identify the approximate origins of the individuals who were not assigned to locations, our next step was to evaluate if these individuals could be assigned to one of the three broader clusters described above (Fig. 2). Assignment to clusters allowed the detection of the possible region of origin of 6 additional individuals, one showed a value very close to the threshold level (69.5%), and 1 remained unassignable (Table 2).

We assigned more than half of the confiscated individuals (14) to 1 of the 3 clusters (F-Ch-C). Four were assigned to the P-PR cluster and 3 to the M-RU cluster.

There was a great deal of variation in the extent to which individuals housed at particular rehabilitation centre actually came from nearby clusters. Specifically, the centre Güirá-Oga, in Misiones province, housed only 2 individuals from the nearby cluster (M-RU where they were reintroduced), while 11 belonged to the cluster F-Ch-C and other 4 to the P-RP clusters (Table 2). Of the 5 confiscated animals housed at Esmeralda, only 4 could be assigned to clusters. Three of them likely came from the nearby F-Ch-C cluster and one from the M-RU cluster. The confiscation sites of these 5 individuals were registered, and the animals that clustered in F-Ch-C (individuals Esmeralda 2, and 4, Supplementary Table S1) were confiscated in Santa Fe province, while the other two (Esmeralda 1 and 5) were confiscated in provinces that do not belong within the natural distribution of *A. caraya* (Supplementary Table S1). Regarding the 3 corpses found in cities, we could assign one that belonged to a cluster in the same region of Argentina where the city lies, and another one that belonged to a different cluster (F-Ch-C, Table 2). In summary, of all 25 analysed individuals, 15 were inferred to have come from sites within the F-Ch-C cluster.

Finally, of the 17 individuals reintroduced (12 in Misiones and 5 in Santa Fe) only four were reintroduced into a site in their cluster of origin (1 into Misiones, 3 into Santa Fe, Table 2, Supplementary Table S1).

## Discussion

Molecular genetic studies have allowed the identification of species confiscated from the illegal wildlife trade, as well as traded animal, timber, and wood products. These applications are even used for species identification from limited samples of body parts (e.g., teeth, feathers, processed tusks), allowing a reliable assessment of the effects of exploitation and the conservation needs of species that would be impossible otherwise. This is the case of the genetic assignment of 28 ivory samples from different elephant populations in Africa^14^ between 1996 and 2014 that resulted in the identification of two major poaching hotspots, or determination of the species of dried shark fins being sold in Asian and Mediterranean commercial markets^40^, allowing the monitoring of trade for conservation assessment. Moreover, a study conducted in South Africa revealed that products labelled as “game meat” belonged to domestic species in 76.5% of cases^41^. Thus, molecular analyses are helpful for poaching detection, traffic route identification, and other crimes involving wildlife.

In a study on tortoises^12^, where researchers also developed a GRDB and correctly assigned 90% of the individuals in that database to their population of origin, the lack of assignment of confiscated individuals was attributed to the fact that they came from different locations than the sampling sites included in the GRDB. Nevertheless, the researchers were able to determine that all the confiscated individuals came from the same population. This other approach of genetic assignment of living animals (mostly confiscated or in captivity) shows how genetic tools can be used by wildlife managers to identify the most probable regions of origin of individuals as well as to determine the genetic appropriateness of potential recipient populations when designing reintroduction projects. Translocations have been used to mitigate population decline and restore locally extinct populations^6^. In these cases, genetic data are necessary to guide the selection of populations of origin to which translocated individuals should be released and subsequently evaluate the success of the restoration^2,42,43^.

In this study, the first application of the GRDB of howler monkeys, our results indicate that the most likely origins of most of the confiscated and surrendered individuals were from the areas around Locs 2 and 3, close to the Argentina-Paraguay border (Fig. 1). Therefore, the largest number of illegally trafficked *A. caraya* originated in this area.

This area is also the location of northeastern Argentina’s largest cities, Chaco and Corrientes, and National Highway 12, the main highway connecting these cities with Buenos Aires. The illegal sale of *A. caraya* has been reported at several locations along this highway^21,22^. This information supports a possible animal trafficking route that begins in northeastern Argentina and ends in Buenos Aires, where the majority of confiscations occur (10 of 22, Supplementary Table S2^21,22^). Importantly, most of the confiscations and surrenders occurred in cities outside the normal distribution of the species (17 of 22, Supplementary Table S2), indicating that these animals are not only opportunistically captured by locals, but that these animals are intentionally transferred to urban centres. This example illustrates how genetic analysis helps trace wildlife trafficking routes and hotspots and thus aids in the planning and implementation of more effective control measures.

On the other hand, 15 of the 17 animals that arrived at the rescue centre Güirá-Oga, in Misiones, were assigned to either to the F-Ch-C or P-RP clusters; twelve of these individuals were subsequently reintroduced near this rescue centre on Isla Palacio, where the endemic genetic variation belongs to the M-RU cluster; thus, translocation and reintroduction resulted in the injection of genetic variation from animals belonging to different genetic clusters. The 5 individuals that arrived at Esmeralda were also reintroduced in a protected area of General Obligado, Santa Fe (Fig. 1). Although nearby localities are not sampled in the database, we would expect that of our sample areas, genetic variation in the liberation area would be most similar to F-Ch-C, similar to the southernmost area of the distribution of *A. caraya*. Of these five reintroduced individuals, three belonged to the same cluster and only one belonged to the M-RU cluster; therefore, translocation and reintroduction of these animals also introduced non-local genetic variability, albeit in a lower proportion.

Our findings highlight the importance of conducting genetic studies prior to the liberation of rescued animals. These results also raise the concern of establishing rehabilitation centres servicing each of the three described clusters that could be considered as management units for *A. caraya* if the goal is to reintroduce animals into their native populations. Based on this, future work could consist of the genetic assignment of all the individuals that are going to be part of translocation programs, as well as the extension of the GRDB in areas where releases are scheduled. In this way, the genetic variability that would be entering a reintroduction site could be evaluated and possible restoration analysis would be possible afterwards.

Conservation genetics is generally not yet well integrated with other efforts in conservation policies. In Latin America, the practical application of genetic principles for the management of threatened species and in the development and implementation of conservation plans should be emphasized^7^. One possible explanation for this disconnect may be that knowledge obtained from scientific research is often not communicated effectively to the field practitioners and/or the authorities who formulate and enact policies.

As shown in the present study, concrete and measurable genetic data represent a very effective tool to help establish and enforce adequate legislation to curb the loss of biodiversity, generate conservation guidelines, and develop population management strategies that include translocation and reintroduction projects.

## Methods

### Sampling for GRDB

The GRDB for *A. caraya* used in this study was built using a previously complied database for many locations in Argentina that contained 143 individuals^9^. The number of individuals in the initial database was increased by adding individuals sampled during a monitoring program for the epidemiological surveillance of YFV and other arboviroses in NHP from several sites in Misiones province in 2017 and 2018 (Table 1 and Fig. 1). We sampled 34 individuals from five newly sampled localities (Locs 11 to 15 in Fig. 1) as well as three individuals from Loc 8 and five individuals from Loc 9, which were resampled localities. In total, these 42 individuals sampled corresponded to 39 new individuals (3 wound up being duplicates of previously sampled in the resampled localities, 1 individual in Loc 8 and 2 in Loc 9; Table 1). Summarizing, we used 178 individuals sampled from 15 localities in the overall GRDB. This included 139 from our previous study^9^ (we excluded 4 individuals from 2 localities that shared an allele at each locus and were thus considered first-order relatives) and 39 new individuals. Table 1 summarizes the number of individuals, geographical coordinates of sampling locations, and type of samples analysed.

### Sampling for genetic assignments

Twenty-five samples were collected for individual genetic assignment. We received hair samples from 17 howler monkeys arriving at Güirá-Oga in 2017. Twelve of these individuals were later reintroduced into a protected area in Isla Palacio at 25°53′32″S, 54°24′38″W (Fig. 1). We also received five tissue samples from monkeys arriving at Esmeralda. All these individuals were later reintroduced into a protected area in General Obligado, Santa Fe at 28°00′12.7″S, 59°32′42.09″W (Fig. 1). A detailed description of these individuals is provided in Supplementary Table S2. Finally, we analysed three tissue samples from monkeys found dead by local authorities in Apóstoles, Posadas, and San Antonio, all in Misiones province in northern Argentina (Fig. 1).

### DNA extraction

Two separate faecal samples per individual were stored at room temperature in 50 ml screw-top tubes containing solid NaCl^44^ until DNA extraction (three months to one year later). DNA was extracted from faeces using the QIAamp DNA Stool Mini Kit (QIAGEN, Valencia, USA), according to the manufacturer’s protocols with slight modifications. DNA was extracted from tissue and hair samples using standard SDS/Proteinase K digestion followed by phenol: chloroform (1 to 1 volume ratio) organic extraction and Microcon P-100 counter-dialysis filters^45^.

### Microsatellite amplification

Ten microsatellites, developed for *A. caraya* or other primates and previously used in studies of *A. caraya* population genetics, were amplified from each sample: AC14, AC17, AC45, TGMS1, TGMS2, D8S165, D17S804, LL1118, LL157 and AB07^46–49^. Genotyping PCRs were performed in a final volume of 25 µl using 5–10 ng of DNA template for tissue samples or 5 µl of the extraction pool from stool samples and included 20 mM Tris–HCl, 50 mM KCl, 1.5 mM MgCl_2_, 0.2 mM each dNTP, 1 U of GoTaq DNA polymerase (Promega, Madison, USA), 1 pmol of each forward primer bearing an M13 tail, 4 pmol of each reverse primer, and 4 pmol of M13 labelled with a fluorescent dye (6‐FAM) on its 5′ end following recommendations from previous studies^48,50^. All amplifications were performed in a Gen Amp ABI 9700 machine (Thermo Fisher, Palo Alto, USA). PCR products labelled with different fluorochromes were combined and the amplicons separated by electrophoresis on an ABI PRISM 310 Genetic Analyzer (Thermo Fisher, Palo Alto, USA). Alleles were manually scored by performing a visual inspection of electropherograms after developing the bin panel for each locus in GeneMapper ID-X v. 1.2 (Thermo Fisher, Palo Alto, USA) using HD400-ROX as internal size standard. For DNA extracted from stool samples, PCR and sizing was repeated twice (in the case of a heterozygous genotype call) or four times (in case of a homozygous genotype call) to minimize possible genotyping errors due to allelic dropout^51,52^. We recorded an allele only if it was observed at least twice in different amplifications from the same DNA extract. All amplification assays included negative controls.

### Ethics statement

This study was carried out in strict accordance with Argentinean laws for research on NHP and following the recommendations of ‘Principles for the Ethical Treatment of Primates’ of the American Society of Primatologists (available at: https://www.asp.org/society/resolutions/EthicalTreatmentOfNonHumanPrimates.cfm). We received specific approval to conduct this study by the Consejo Nacional de Investigaciones Científicas y Técnicas (CONICET) of Argentina (no. 11420110100322CO). Sampling permits for all the locations of the original database previously complied were already published^7^. Additional specific sampling permits for the new samples presented in this study were obtained from the Ministry of Ecology, Misiones province, Argentina (Permit number: 9910-00086/17) and from the Ministry of Production Santa Fe province, Argentina (Permit Number: GT 13605). Faecal collection was conducted without capturing animals and therefore does not cause any harm to the studied species.

### Statistical analysis

Genotypes were screened for null-alleles and to discriminate between errors in allele frequency estimates caused by null-alleles, allele dropout or stutter bands using Micro-Checker v2.2.3^32^. Numbers of different alleles, effective and private alleles, observed heterozygosity (H_o_), expected heterozygosity (H_e_), unbiased expected heterozygosity (uH_e_) and inbreeding coefficient were computed with the software GenAlEx v6.5^34^ for each locus and population. Deviations from Hardy-Weinberg equilibrium (HWE) were assessed by employing an exact test and F_IS_ inbreeding coefficient using Arlequin v 3.5 software^33^. Allelic richness was calculated for each locus in a population using the equation: equation:

$$AR=\sum \,\lfloor 1-(\frac{\begin{array}{c}2N-Ni\\ 2n\end{array}}{\begin{array}{c}2N\\ 2n\end{array}})\rfloor ,$$

where *Ni* represents the number of alleles of type *i* among the *2N* genes, and *n* is sample size, using the software, Fstat v2.9.4^53^.

The new complete set of samples collected was analysed using non-spatial Bayesian clustering with the Structure v.2.3.4^35^ program. A series of 20 independent runs per *K* (ranging from 2 to 6) was conducted using the admixture model with correlated allele frequencies, sampling locations as a prior (LOCPRIOR), and 500,000 Monte Carlo-Markov iterations after a burn-in of 50,000 replicates. The data analysis procedure was further refined using Clump software^54^ and a bar plot was constructed with the Disrupt software^55^. The most likely number of *K* was identified using the method described by Evanno^36^.

Assignment tests give the probability of an individual’s multilocus genotype of belonging to reference populations/locations or clusters^56^.

We used Bayesian methods computed by the software GeneClass2^37^ to assign the origin of confiscated individuals of unknown origin into the 15 potential locations sampled and into the different three clusters described here using a leave one-out procedure, excluding self-assignment and being 0.05 the assignment threshold score (or p-value, set by the program for default). After testing all the combinations of approaches presented by the program, we chose the Bayesian criteria described by Rannala & Mountain^57^, resulting in a higher quality index and the highest number of correctly assigned individuals when tested against the database. This program is suitable for our study because it does not assume that all populations of origin have been sampled.

For each individual, the best matching location and cluster were sorted and a score in percentage was obtained. The score of an individual, *i*, in a population, *T*, is computed as follows:

$$Score\,i,T=\frac{Li,\,T}{{\sum }_{j=1}^{P}Lj,T},$$

with *Li*, *T* the likelihood value of the individual *i* in the population *T*.

Once this probability of assignment of each individual to a certain location or cluster was obtained, we established a threshold value above which assignment was considered reliable. The criteria were that the scoring for a given population was superior to 70%. This threshold was based on our correctly obtained re-assignments of 73% of the database individuals to their locality and in previous works on assignments for fishes and birds^33,39^.

## Supplementary information

Supplementary Tables.

## Data Availability

The genotypic reference database (GRDB) for *A. caraya* presented in this study is available at: 10.5281/zenodo.3660723.
