# Supplementary material for: Genetic assignment of illegally trafficked neotropical primates and implications for reintroduction programs
Source: Sci Rep. 2020 Feb 28;10:3676. doi: 10.1038/s41598-020-60569-3 (PMC7048725; doi:10.1038/s41598-020-60569-3)
Supplement: Supplementary file 1 — Supplementary Tables. [file 41598_2020_60569_MOESM1_ESM.docx]

**Genetic assignment of illegally trafficked neotropical primates and implications for reintroduction programs.**

**Luciana Inés Oklander*^1^, Mariela Caputo^2^, Agustín Solari^3^ & Daniel Corach^2^**

Supplementary Table S1: Genotypes of twenty-five assigned individuals using ten microsatellites characterized for *A. caraya*^41,42,43,44.^

|  | D17S804 | TGMS2 | AC17 | D8S165 | AC14 | AC45 | AB7 | LL1118 | LL157 | TGMS1 |
| --- | --- | --- | --- | --- | --- | --- | --- | --- | --- | --- |
| Güirá-Oga 1 | 192-192 | 349-349 | 263-263 | 160-160 | 238-238 | 264-264 | 202-202 | 154-154 | 254-264 | 331-331 |
| Güirá-Oga 2 | 192-192 | 341-341 | 258-258 | 160-164 | 238-238 | 268-276 | 202-202 | 152-162 | 266-272 | 331-347 |
| Güirá-Oga 3 | 192-192 | 351-353 | 258-263 | 160-166 | 238-238 | 264-268 | 202-202 | 154-172 | 254-262 | 331-343 |
| Güirá-Oga 4 | 192-192 | 353-353 | 258-258 | 166-166 | 238-238 | 284-284 | 202-202 | 154-182 | 248-266 | 331-331 |
| Güirá-Oga 5 | 180-192 | 351-351 | 258-258 | 160-160 | 238-238 | 276-276 | 202-202 | 152-152 | 260-262 | 343-343 |
| Güirá-Oga 6 | 192-192 | 353-355 | 258-258 | 160-160 | 238-238 | 268-268 | 202-202 | 152-152 | 248-266 | 347-347 |
| Güirá-Oga 7 | 182-192 | 353-353 | 258-258 | 160-160 | 238-238 | 284-284 | 202-202 | 152-152 | 248-266 | 331-331 |
| Güirá-Oga 8 | 182-192 | 341-341 | 258-258 | 160-160 | 238-238 | 292-292 | 202-202 | 160-196 | 248-266 | 343-343 |
| Güirá-Oga 9 | 182-182 | 341-353 | 258-258 | 160-166 | 238-238 | 284-296 | 202-202 | 154-154 | 248-254 | 341-347 |
| Güirá-Oga 10 | 180-192 | 341-353 | 258-258 | 160-164 | 238-238 | 264-268 | 202-202 | 164-192 | 248-264 | 331-343 |
| Güirá-Oga 11 | 192-194 | 353-355 | 258-258 | 160-160 | 238-238 | 280-284 | 204-202 | 152-152 | 262-262 | 331-331 |
| Güirá-Oga 12 | 192-192 | 351-353 | 258-258 | 160-160 | 238-238 | 264-268 | 202-202 | 154-156 | 262-262 | 331-331 |
| Güirá-Oga 13 | 192-192 | 341-353 | 258-258 | 160-160 | 238-238 | 268-280 | 202-202 | 152-152 | 266-266 | 331-343 |
| Güirá-Oga 14 | 182-192 | 351-353 | 258-258 | 160-160 | 238-238 | 268-284 | 202-202 | 154-154 | 264-266 | 331-331 |
| Güirá-Oga 15 | 192-192 | 341-341 | 258-258 | 160-160 | 238-238 | 276-280 | 202-202 | 152-152 | 266-268 | 343-343 |
| Güirá-Oga 16 | 192-194 | 355-355 | 258-263 | 160-172 | 242-242 | 272-300 | 202-202 | 152-184 | 248-248 | 331-331 |
| Güirá-Oga 17 | 192-192 | 355-355 | 258-258 | 166-166 | 238-238 | 284-284 | 202-202 | 154-180 | 248-264 | 331-331 |
| Esmeralda 1 | 192-192 | 353-353 | 258-258 | 164-166 | 238-238 | 264-272 | 202-202 | 152-192 | 254-254 | 331-341 |
| Esmeralda 2 | 182-192 | 341-353 | 258-258 | 160-160 | 238-238 | 264-268 | 202-202 | 154-192 | 266-266 | 331-343 |
| Esmeralda 3 | 192-192 | 341-353 | 258-258 | 160-160 | 238-238 | 272-284 | 202-202 | 152-182 | 254-264 | 343-343 |
| Esmeralda 4 | 192-192 | 341-341 | 258-258 | 160-160 | 238-238 | 0 | 202-202 | 154-194 | 248-266 | 343-347 |
| Esmeralda 5 | 192-192 | 353-353 | 258-263 | 160-160 | 238-238 | 0 | 202-202 | 190-196 | 254-254 | 331-331 |
| Found dead in Pop 13, Misiones | 192-192 | 341-353 | 258-258 | 164-166 | 238-238 | 264-272 | 202-202 | 154-188 | 266-268 | 331-347 |
| Found dead in Posadas, Misiones | 192-192 | 349-353 | 258-258 | 160-172 | 238-240 | 272-272 | 202-202 | 154-178 | 248-266 | 331-331 |
| Found dead in San Antonio, Misiones | 184-194 | 353-357 | 258-263 | 160-160 | 238-240 | 268-268 | 202-202 | 152-152 | 254-266 | 331-343 |

Supplementary Table S2: Details on individuals that were confiscated and surrendered to rescue centres. Individual’s age was estimated from their arrival at the rescue centres by sexual maturity characteristics and in case of mature individuals by the preservation condition of their teeth. SGAyDS: Secretaría de Gobierno de Ambiente y Desarrollo Sustentable de la Nación Argentina, MEyRNR Misiones: Ministerio de Ecología y Recursos Naturales Renovables de la Provincia de Misiones, MPSF: Ministerio de la Producción de la Provincia de Santa Fe, RN Corrientes: Recursos Naturales de la Provincia de Corrientes, DF S. Estero: Dirección de Fauna de la Provincia de Santiago del Estero.

|  | number | | Name | Sex | Age | Conf/Surr | Conf/Surr Site | Authority | Reintroduced | Conf/Surr Site is within the natural distribution |
| --- | --- | --- | --- | --- | --- | --- | --- | --- | --- | --- |
| Güirá-Oga 1 | | GT4897 | Nika | Female | 4/5 | Surrendered | Gran Buenos Aires, Buenos Aires | SGAyDS | Yes | No |
| Güirá-Oga 2 | | GT4897 | Olo | Male | 5/6 | Confiscated | Gran Buenos Aires, Buenos Aires | SGAyDS | Yes | No |
| Güirá-Oga 3 | | GT11014 | Chiqui | Male | 4/5 | Confiscated | Gran Buenos Aires, Buenos Aires | SGAyDS | Yes | No |
| Güirá-Oga 4 | | GT11014 | Candi | Female | 4/5 | Confiscated | Gran Buenos Aires, Buenos Aires | SGAyDS | Yes | No |
| Güirá-Oga 5 | | AE1139 | Monchi | Female | 5/6 | Surrendered | Posadas, Misiones | MEyRNR Misiones | Yes | Yes |
| Güirá-Oga 6 | | AE1068 | Trini | Female | 7/8 | Surrendered | Corrientes, Corrientes | RN Corrientes | Yes | Yes |
| Güirá-Oga 7 | | GT2551 | Tucu | Male | 4/5 | Confiscated | Unknown | DF S. Estero | Yes | No |
| Güirá-Oga 8 | | GT2551 | Guardian | Male | 6/7 | Confiscated | Unknown | DF S. Estero | Yes | No |
| Güirá-Oga 9 | | GT2551 | Rubi | Female | 6/7 | Confiscated | Unknown | DF S. Estero | Yes | No |
| Güirá-Oga 10 | | GT2551 | Ayo | Male | 3 | Confiscated | Unknown | DF S. Estero | Yes | No |
| Güirá-Oga 11 | | GT1854 | Cesar | Male | 9/10 | Confiscated | Gran Buenos Aires, Buenos Aires | SGAyDS | Yes | No |
| Güirá-Oga 12 | | GT4594 | Peque | Male | 2 | Confiscated | CABA, Buenos Aires | SGAyDS | Yes | No |
| Güirá-Oga 13 | | GT4594 | Danette | Female | 6/7 | Confiscated | Gran Buenos Aires, Buenos Aires | SGAyDS | No | No |
| Güirá-Oga 14 | | GT4594 | Poju | Male | 13/14 | Confiscated | Gran Buenos Aires, Buenos Aires | SGAyDS | No | No |
| Güirá-Oga 15 | | GT4594 | Budi | Male | 14/15 | Surrendered | CABA, Buenos Aires | SGAyDS | No | No |
| Güirá-Oga 16 | | AE1070 | Fido | Male | 4/5 | Surrendered | Posadas, Misiones | MEyRNR Misiones | No | Yes |
| Güirá-Oga 17 | | GT8225 | Adul | Male | 5/6 | Surrendered | Gran Buenos Aires, Buenos Aires | SGAyDS | No | No |
| Esmeralda 1 | | GT13605 | Susana | Female | 15 | Confiscated | Salto Grande, Entre Ríos | MPSF | Yes | No |
| Esmeralda 2 | | GT13605 | California | Female | 3/4 | Confiscated | San José del Rincón, Santa Fe | MPSF | Yes | No |
| Esmeralda 3 | | GT13605 | Vera | Female | 4/5 | Confiscated | Vera, Santa Fe | MPSF | Yes | Yes |
| Esmeralda 4 | | GT13605 | Ocampo | Male | 2 | Confiscated | Villa Ocampo, Santa Fe | MPSF | Yes | Yes |
| Esmeralda 5 | | GT13605 | Amaro | Male | 3/4 | Confiscated | San Luis, San Luis | MPSF | Yes | No |
